# A microarray study of MPP^+^-treated PC12 Cells: Mechanisms of toxicity (MOT) analysis using bioinformatics tools

**DOI:** 10.1186/1471-2105-6-S2-S8

**Published:** 2005-07-15

**Authors:** Zengjun Xu, Tucker A Patterson, Jonathan D Wren, Tao Han, Leming Shi, Helen Duhart, Syed F Ali, William Slikker

**Affiliations:** 1 Division of Neurotoxicology, National Center for Toxicological Research, U.S. Food and Drug Administration, 3900 NCTR Road, Jefferson, Arkansas 72079, USA; 2 Advanced Center for Genome Technology, Department of Botany and Microbiology, 101 David L. Boren Blvd., The University of Oklahoma, Norman Oklahoma 73019, USA; 3 Division of Systems Biology, National Center for Toxicological Research, U.S. Food and Drug Administration, 3900 NCTR Road, Jefferson, Arkansas 72079, USA

## Abstract

**Background:**

This paper describes a microarray study including data quality control, data analysis and the analysis of the mechanism of toxicity (MOT) induced by 1-methyl-4-phenylpyridinium (MPP^+^) in a rat adrenal pheochromocytoma cell line (PC12 cells) using bioinformatics tools. MPP^+ ^depletes dopamine content and elicits cell death in PC12 cells. However, the mechanism of MPP^+^-induced neurotoxicity is still unclear.

**Results:**

In this study, Agilent rat oligo 22K microarrays were used to examine alterations in gene expression of PC12 cells after 500 μM MPP^+ ^treatment. Relative gene expression of control and treated cells represented by spot intensities on the array chips was analyzed using bioinformatics tools. Raw data from each array were input into the NCTR ArrayTrack database, and normalized using a Lowess normalization method. Data quality was monitored in ArrayTrack. The means of the averaged log ratio of the paired samples were used to identify the fold changes of gene expression in PC12 cells after MPP^+ ^treatment. Our data showed that 106 genes and ESTs (Expressed Sequence Tags) were changed 2-fold and above with MPP^+ ^treatment; among these, 75 genes had gene symbols and 59 genes had known functions according to the Agilent gene Refguide and ArrayTrack-linked gene library. The mechanism of MPP^+^-induced toxicity in PC12 cells was analyzed based on their genes functions, biological process, pathways and previous published literatures.

**Conclusion:**

Multiple pathways were suggested to be involved in the mechanism of MPP^+^-induced toxicity, including oxidative stress, DNA and protein damage, cell cycling arrest, and apoptosis.

## Introduction

DNA microarrays have been increasingly applied as a tool for the simultaneous monitoring of relative expression levels of thousands of genes for samples under various conditions, e.g., normal versus disease, and control versus drug or toxicant treatment [1-3], and offer a promising means to better understand how cells react to environmental perturbations. Their popularity, in part, is reflected by the number of microarray-related publications indexed in PubMed, which have been increasing exponentially. With the improvement of microarray chip quality and tools for data quality assurance, the focus in this field has gradually switched from determination of the gene numbers with altered expression levels to the analysis of the biological mechanism by categorizing significantly changed genes into functional groups and pathways [5-7]. Toxicogenomics, a emerging field combining genomics and bioinformatics to identify and characterize mechanisms of toxicity (MOT) for drugs and various compounds, has been developed quickly during the last several years [8-13]. Microarrays, with their power to examine genome-wide transcriptional responses, have become a key technology in toxicogenomics.

During the last two decades, MPTP (1-methyl-4-phenyl-1,2,5,6-tetrahydropyridine) – induced neurotoxicity has attracted a great deal of attention because of the similarity of its toxic effects to the biochemical changes in the brains of patients with Parkinson's disease (PD) [14,15]. PD is a progressive neurodegenerative disorder that results in degeneration of dopaminergic neurons in the substantia nigra (SN) and dopamine depletion in the striatum [16]. Although MPTP does not exactly reproduce PD, it has been an extremely valuable tool to model many features of PD in animals, and has led to a better understanding of crucial aspects of the sub-cellular events participating in the evolution of the PD clinical syndrome and neurotoxicity [17-20]. In order to be active, MPTP requires monoamine oxidase B to be converted into MPP^+ ^(1-methyl-4-phenylpyridinium) [21]. MPP^+ ^is selectively taken up by dopaminergic neurons via the dopamine transporter of the plasma membrane [22,23] and produces neuronal loss in substantia nigra (SN), striatal dopamine (DA) depletion and behavioral impairments in humans [24], primates [14], and mice [15,25-28].

PC12 cells, a rat clonal pheochromocytoma cell line [29], possess dopamine synthesis, metabolism and transporter systems [30-32], and therefore have been used as a model for studies of MPP^+ ^neurotoxicity and PD. A plethora of evidence has demonstrated that MPP^+ ^depletes dopamine and elicits cell death in PC12 cells [33-36]. Previously, we demonstrated that MPP^+^-induced DA depletion and cell loss in PC12 cells is dose and time-course responsive [37]. However, the mechanism of MPP^+ ^neurotoxicity in PC12 cells is still unclear. Generally, it is believed that MPP^+ ^directly and/or indirectly inhibits mitochondrial complex I, causing abnormal energy metabolism and increased production of reactive oxygen species (ROS), resulting in cell death [34,36,38]. Our study indicated that MPP^+ ^may compromise heat shock protein (HSP) cell defense systems and cause apoptosis in NGF-differentiated PC12 cells and C56, but not CD1, mice [37,39]. However, some studies have suggested that the effect of MPP^+ ^on PC12 cells might be independent of ROS [35,40]. Recent studies have also demonstrated that endoplasmic reticulum [41], PI-3 [42] and cAMP pathways [43] may be possible targets for MPP^+^-induced neurotoxicity. Thus, MPP^+ ^may cause PC12 cell injury and death via multiple complex mechanisms. Therefore, DNA microarray analysis, by measuring the expression of large numbers of genes, is a promising tool to help elucidate the mechanism of MPP^+^-induced toxicity in PC12 cells.

With the development of bioinformatics, various toxicogenomic databases and bioinformatics tools are available for data manipulation and mining [9,44,45]. The Center for Toxicoinformatics in the FDA's National Center for Toxicological Research (NCTR/FDA) has developed a public microarray data management and analysis software, called ArrayTrack, for FDA-wide microarray data storage and preliminary data analysis [10,46]. In this study, alterations of gene expression were examined using the Agilent microarray platform and the data were analyzed using ArrayTrack and a software package called IRIDESCENT.

## Methods and materials

### Cell culture and MPP^+ ^treatment

PC12 cells at passage 15–20 (ATCC, Manassas, VA) were grown in 75 cm^2^tissue culture flasks at 37°C under an atmosphere of 5% CO_2_/95% air in RPMI 1640 medium (Sigma, St. Louis, MO) containing 10% horse serum and 5% fetal bovine serum (complete media). At about 80% confluence, cells were placed into 24-well plates and cultured for another 2 days, and then were treated with 500 μM MPP^+ ^iodide (Sigma, 100% purity by HPLC), a dose that induces significant decrease in dopamine content and cell viability [37].

### RNA Isolation

Twenty-four hours after MPP^+ ^treatment, 12 wells of PC12 cells from same treatment group in a 24-well plate were pooled into one single sample. Two control and two MPP^+^-treated samples were prepared for microarray hybridization. Total RNA was extracted using Qiagen™ RNeasy Mini Kits (Qiagen, Valencia, CA) following the RNeasy mini protocols for isolation from animal cells (I. spin protocol). RNA quantity was measured using the NanoDrop^® ^ND-1000 UV-Vis Spectrophotometer (Wilmington, DE), while RNA quality was monitored using Agilent 2100 bioanalyzer and expressed as RNA integrity number (RIN) value using Agilent 2100 RIN Beta Version Software (Palo Alto, CA). For all 4 RNA samples, the ratios of 260/280 (indication of protein contamination) and 260/230 (indication of reagent contamination) determined by the NanoDrop spectrophotometer were above 2, and the RIN values determined by an Agilent bioanalyzer were 10 (Best RNA quality according to the grading of Agilent 2100 RIN Software).

### Microarray studies

Agilent 22K 60-mer oligonucleotide microarray slides (Palo Alto, CA) were used and a dye-swap experimental design was applied. High-quality RNA samples (200 ng each) acquired from PC12 cells were amplified and labeled with Cy5-and Cy3-CTP (Amersham Biosciences) to produce labeled cRNA using Agilent low RNA input fluorescent linear amplification kits following the manufacturers protocol. After the amplification and labeling, the dye-incorporation ratio was determined using a Nanodrop spectrophotometer and the ratios were within 10 to 20 pmol per μg cRNA, the range the manufacturer suggests prior to hybridization. For hybridization, the Agilent 60-mer oligo microarray processing protocol (Rev. 7, SSC Wash/6-screw hybridization chamber) was strictly followed. Briefly, 750 ng Cy3-labeled control and 750 ng Cy5-labeled MPP^+^-treated sample were mixed and incubated with an Agilent microarray slide for 17 hours using an Agilent *in situ *hybridization kit following SSC buffer washing. Sample pairs were dye-swapped and processed at the same time. The washed slides were immediately dried using an ultra pure filtered N_2 _stream in an ozone-free Biobubble. After drying, the slides were scanned using an Axon GenePix 4000B scanner with the PMT settings at 770 for Cy5 and 670 for Cy3, and the raw data were generated using GenePix Pro 6.0 software (Axon Instruments, Union City, CA).

### Data analysis and MOT interpretation

**Figure 1:**
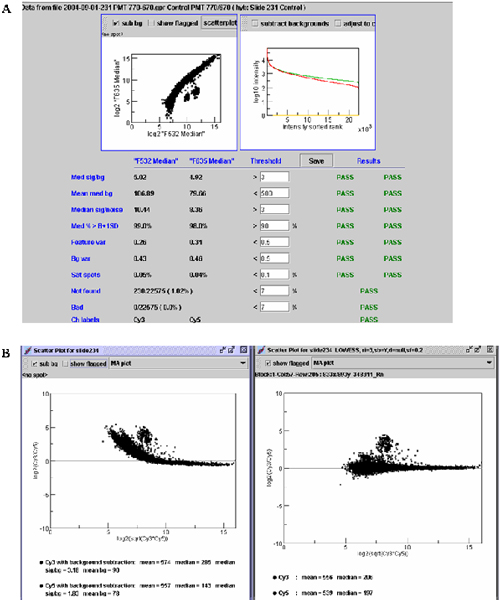
Data quality control and Lowess normalization. A. the graph shows a sample of data quality controlled by ArrayTrack. The lower part of Figure 1A showed the criterion parameters, the values of Cy3 (F532) and Cy5 (F635), default threshold, and if the specific slide passed the threshold. The upper part shows the scatter plot of the two dyes (Cy3 and Cy5) (Left) and the spot density distribution (right). B. Lowess normalization. The left and right panel shows the MA plot before and after the normalization, respectively.

Four datasets were acquired in this experiment, Slide 230: control 1 (Cy3)/treated 1(Cy5); Slide 231: control 2(Cy3)/treated 2(Cy5); Slide 232 (dye-swap of slide 230): control 1 (Cy5)/treated 1 (Cy3); Slide 233 (dye-swap of slide 231): control 2(Cy5)/treated 2(Cy3). Raw data generated from GenePix Pro 6.0 software was input into the ArrayTrack database. ArrayTrack is logically constructed of three linked components: a) a database (MicroarrayDB) that stores microarray experiment information; b) tools (TOOL) for data visualization and analysis; and c) libraries (LIB) that provide curated functional data from public databases for data interpretation. Specifically, ArrayTrack is MIAME (Minimum Information About A Microarray Experiment) supportive for storing both microarray data and experiment parameters associated with a toxicogenomics study. A quality control mechanism is implemented to assess the quality of entire expression data as well as quality of each spot on the chip. In addition, many data analysis and visualization tools are available with ArrayTrack, including four normalization methods, several statistical approaches for identification of differentially expressed genes, clustering/classification methods. ArrayTrack also provides a rich collection of functional information about genes, proteins and pathways drawn from various public biological databases for facilitating data interpretation [10,46]. After data input, an automated data quality control was made by serial criteria of data quality control parameters in ArrayTrack to assure the data quality (Fig. 1A). Subsequently, data were normalized using the Lowess normalization method [47] to correct intensity-dependent ratio bias in ArrayTrack (Fig. 1B). Raw intensity data were logarithm (base 2) transformed and the log ratio of treated/control was calculated in ArrayTrack. The data set was then exported and the subsequent data analysis was performed employing JMP statistical software 5.0.1 and Spotfire DecisionSite bioinformatics software. The genes were categorized based on their functions derived from OMIM  and PubMed literature  as well as GO biological process and molecular functions.

### IRIDESCENT

A software package called IRIDESCENT [48-50] was also used to tie responding genes back to the published literature and identify commonalities among and between genes, diseases, chemical compounds, ontological categories and FDA-approved drugs. Commonalities are scored based upon how many genes within the entire set of microarray responders are observed to be related to each ''object'' via their co-occurrence within MEDLINE abstracts versus how many relationships would be expected statistically, by chance[49]. This enables validation of experimental data by comparing the microarray response to the published literature to verify that at least some previously documented relationships are being reproduced by the experiment. It also enables the identification of common themes and potential new leads. Graphs were drawn using GraphViz, a software package written by AT&T Labs.

## Results

### Microarray data consistency

**Figure 2:**
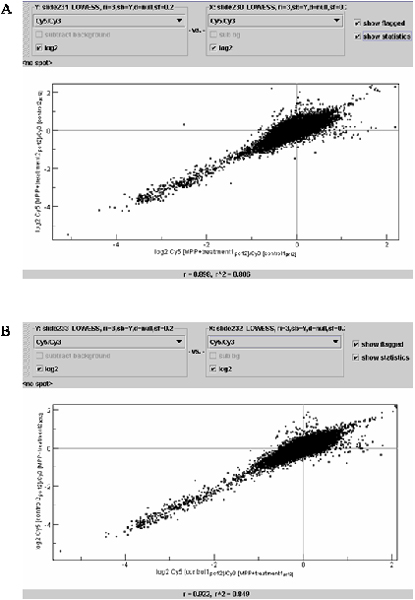
Pearson correlation ratio between biological repeats determined by mixed scatter plot tool in ArrayTrack. A. correlation of slide 230 (treated/control sample pair 1)/231 (treated/control sample pair 2); B. correlation of slide 232/233. It should be noted that all the Agilent embedded positive and negative controls were included.

**Figure 3:**
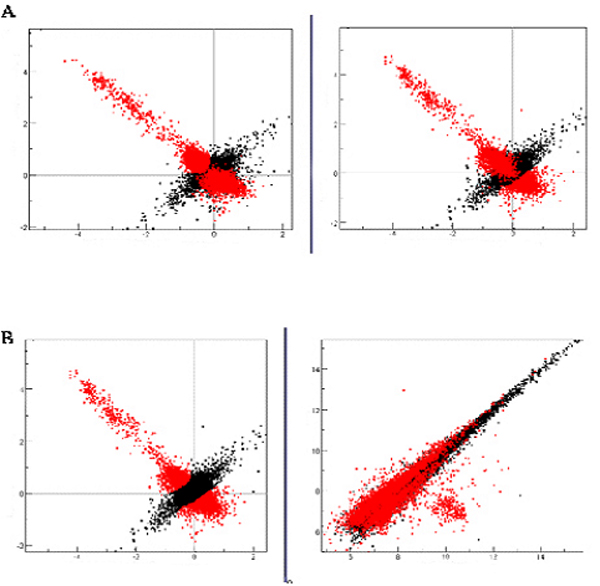
Anti-correlation spots were determined in ArrayTrack. A. Determination of the consistency of anti-related spots in different dye-swapped slide sets. The left panel shows the scatter plot of slide 230/232 and the right shows the scatter plot of slide 231/233. Red color labeled the same spots in the two graphs. B. Determination of the intensity of anti-related spots. The left panel shows the anti-correlation effect of slide 231/233 and the right one shows the intensity scatter plot of Cy5 and Cy3 of each spot on slide 231. Red color labeled represents the anti-correlated spots (left) and their corresponding log intensities (right). It should be noted that slide 231 is used as an example to show the intensities of anti-correlated spots, but similar results are observed in all other 3 slides.

Data consistency was examined employing both ArrayTrack tools and JMP software. Figure 2 shows the correlation ratios between sample pairs (r = 0.898 for slide 230 versus 231; r = 0.922 for slide 232 versus 233), indicating data consistency between biological samples. However, poor correlation ratios of dye-swapped slides (Slide 230/232 and slides 231/233) were observed in this study (Fig. 3) due to anti-correlation, a common phenomenon in dye-swapped slides. Further analysis using ArrayTrack tools indicated that these spots are consistent in the two swapped-slide sets (shown red) (Fig. 3A) and most of them are low-intensity spots and internal Cy3 controls (shown red) (Fig. 3B).

### Identification of genes altered by MPP^+ ^treatment

**Figure 4:**
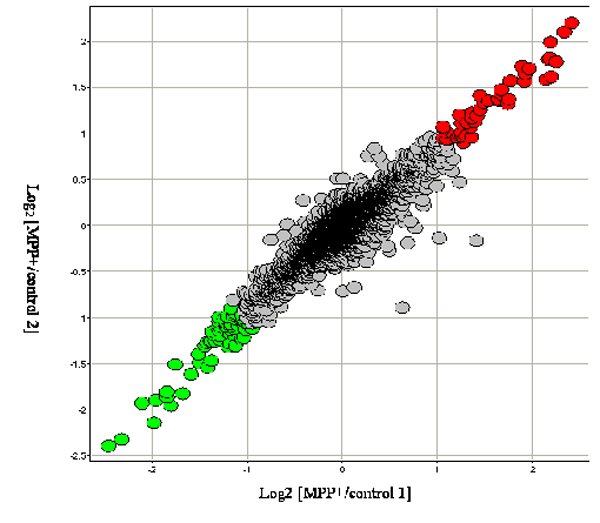
Scatter plot of the averaged log ratio of dye-swapped slide sets (Avg [230+232] / Avg [231+233]) in Spotfire (Correlation ratio: R = 0.90). Red labeled spots represents those with log values ≥ 1 (2-fold and above increase) and green labeled spots represents those with log values ≤ -1 (2-fold and above decrease).

Genes with the lowest average intensity in this study (an arbitrary threshold at log 2 transformed intensity = 8) were excluded to reduce variance. Log ratios of the dye-swapped slides were averaged and the mean of two sample pairs were subsequently used to determine the fold change of gene expression in MPP^+^-treated groups in contrast to control. Figure 4 shows the correlation of the treated/control log (base 2) of the two sample pairs after the dye-swapped slides were averaged (r = 0.90), indicating low variance between the two samples sets. In this study, a 2-fold cutoff was applied as significant. Genes with log 2 transformed ratio (MPP^+ ^treated/control) = 1 (representing 2-fold and above increase) showed red and ≤ 1 (representing 2-fold and above decrease) showed green. In total, there were 106 genes (44 genes induced and 62 genes repressed) with equal to and greater than a 2-fold change with MPP^+ ^treatment.

### MOT analysis

Based on GO term information, OMIM and PubMed literature, ArrayTrack was used to categorize genes into 6 functional groups, which were: Oxidative stress, DNA and protein damage, metabolic process, neurotransmission and neuronal growth, cell arrest and apoptosis, and cell cycle (Tables 1, 2, 3, 4, 5, 6). Additionally, genes that cannot be categorized or with unclear functions were also listed (Table 7).

### Genes associated with oxidative stress (Table 1)

Nine genes were functionally categorized in this group. Vmp1 is a stress-induced gene which may promote formation of intracellular vacuoles followed by cell death [51]. Although it is still not clear the exact function of isg12(b), it is believed to play a role in resisting cellular or environmental stress. Herpud1 encodes an endoplasmic reticulum stress-inducible protein [52]. Heat shock protein is generally induced by oxidative stress. Ephrin A1 acts as an early response protein in response to oxidative stress. This gene along with its receptor increased in response to DNA damage and is regulated by p53 in apoptosis [53]. Ftl1 is a gene that is often induced due to iron-related oxidative stress, which is important for Parkinson's disease [54]. The induction of these genes indicates that MPP^+ ^may induce oxidative stress in PC12 cells. However, leukotriene B4 12-hydroxydehydrogenase which also plays a role in antioxidative function [55], CD24 involved in defense response [56] and Gstm1, a general stress-response gene, were down-regulated in response to MPP^+ ^treatment, an outcome in opposition to the enhanced oxidative stress hypothesis. However, it should be noted that the above 3 genes are also involved in other functions. Alterations of these 3 genes may provide other mechanistic information for MPP^+^-induced toxicity.

### Genes associated with DNA and protein damage (Table 2)

Both Myd116 [57] and Ddit3 (OMIM: +126337) are DNA-damage inducible genes, and their induction provides direct evidence for DNA damage with MPP^+ ^treatment. Dut is involved in purine metabolism, thus, the decrease of this enzyme may compromise the DNA-repair system. In addition, as mentioned above, increased expression of ephrin A1 is also an indication of DNA damage. Limited evidence was found to indicate protein damage. Tryptophanyl-tRNA synthetase catalyzes the first step of protein synthesis and is an essential function in the cell's protein synthesis machinery (OMIM: *191050). Cathepsin L is a protein associated with proteolysis and peptidolysis and induced by oxidative stress [58,59]. These genes, along with heat shock protein 70, indicate cellular response to protein damage. Tcp1 functions as a cytosolic chaperone in the biosynthesis of tubulin [60,61]. Although there is no evidence to indicate that this protein is involved in protein damage and repair, it has been reported that tubulin is involved in MPP^+ ^elicited toxicity in PC12 cells [62]. The decreased expression of this gene may affect the synthesis of tubulin, whose assembly into microtubules is critical to many cellular processes. It should be noted that LOC296368 has a sequence similar to ubiquitin-conjugation enzyme E2C, and is down-regulated with MPP^+ ^treatment. However, because the exact function of this sequence is still unknown, the effect of these data on our hypothesis is unclear.

### Genes associated with metabolic processes (Table 3)

If cellular functions are severely damaged, it is expected that most metabolic activities, especially fuel-utilization and energy metabolism, will be compromised. As Table 3 shows, MPP^+ ^induces suppression of chromogranin A, a gene that modulates glucose, lipid and protein metabolism, and other genes associated with energy production, except acsl4. Therefore, we can primarily conclude that metabolic processes were down-regulated by MPP^+ ^in PC12 cells. In addition, chromogranin A is also co-stored and co-released with catecholamines from storage granules in the adrenal medulla (OMIM: *118910). Although the function is still not known, the decrease of chromogranin A may indicate decreased dopamine content with MPP^+ ^treatment as previously reported [33,37].

### Genes associated with neurotransmission and neuronal growth (Table 4)

MPP^+^-induces dopamine depletion, however, our preliminary study using real-time PCR has demonstrated that there are only minor gene expression changes with MPP^+ ^treatment in PC12 cells (data not shown), unlike that *in vivo *[28]. In this study, instead of tyrosine hydroxylase, dopa decarboxylase (Ddc) was down-regulated following MPP^+ ^treatment. In addition, cytochrome b561, a major transmembrane protein that is specific to catecholamine and neuropeptide secretory vesicles of the adrenal medulla (OMIM: *600019), was down-regulated. As mentioned above, chromogranin A may be involved in dopamine metabolism in PC12 cells.

In PC12 cells, genes involved in neurogenesis and neuronal development were down-regulated following MPP^+ ^treatment. These include Vgf (OMIM: *602186), Nnat [63], Plk2 [64], Hmgb2 (GO terms, biological process) and Bhlhb3 (GO terms, biological process). The decrease of these growth-related genes may suggest cell damage, cell growth arrest and a tendency toward apoptosis.

### Genes associated with general cell growth arrest and apoptosis (Table 5)

With MPP^+ ^treatment,, the expression of Gadd45a and Gas5 [65], genes associated with cell growth and responsive to DNA-damage stimulus [66], were induced. As shown above, induction of ephrin A1 also indicates p53-dependent activation in response to DNA damage [53]. Furthermore, Trib3 (Kegg pathway) and Vmp1, which are involved in apoptosis, are both induced. Adenosine A2a receptor is also included in this group because it has been reported that Activation of A2A adenosine receptors (Adora2a) was found to prevent reactive oxygen species (ROS) formation and apoptosis in pheochromocytoma PC12 cells [67]. The repression of this gene may facilitate apoptosis and ROS damage. In addition, this receptor is cAMP mediated, thus the repression of this receptor may be involved in subsequent cAMP regulated functions, such as CREB. ADM is one of the proteins that are cAMP pathway regulated. Although the function is still not clear, it has been reported to be highly expressed in pheochromocytoma and adrenal medulla (OMIM: *103275).

### Genes associated with cell-cycle (Table 6)

It is evident that if cell growth is arrested, cell cycle and proliferation should be ceased. Thus, it is not surprising to see that genes for anti-proliferation (Btg1, Copeb) were induced and genes for cell cycle and replications were reduced. As shown in Table 6, Mcmd6 (mini chromosome maintenance deficient 6), which belongs to a family of early S-phase proteins required for DNA replication, may play a role in cell-cycle progression and DNA replication [68]. Calcyclin (S100A6) is a calcium-binding protein that belongs to the family of S100 proteins. Its gene was discovered on the basis of its cell cycle-dependent expression. This gene is expressed at its maximal level during the transition between G_0 _to S phase of the cell cycle [69,70]. Snf1lk encodes a protein kinase that may function in cell cycle regulation [71]. Tcf19 is a transcription factor that may be involved in cell growth [72]. H2Afz is a protein synthesized throughout the cell cycle (OMIM: *142763). These genes, along with some typical genes of the cell cycle (cdc2a, S-phase kinase-associated protein, Rfc2, Ret, Pcna), were all down-regulated by MPP^+ ^treatment. Although the oncogene c-Myc has been considered a gene for proliferation, it is usually up-regulated during apoptosis [73,74]. Thus, the induction of this gene is consistent with apoptotic processes induced by MPP^+^.

### Genes with unknown functions (Table 7)

There are also some genes that cannot be categorized into gene groups. Generally this is due to their unknown functions. However, GPNMB, expressed in melanoma cell lines, was preferentially expressed in low-metastatic cell lines (OMIM: *604368). There was an inverse relationship between the expression of GPNMB compared to calcyclin (OMIM: *604368). In PC12 cells, this pattern was also observed. Acp5 is a gene related to bone growth (OMIM: *171640), which made it difficult to correlate with its function in PC12 cells.

### IRIDESCENT Analysis

Thus far, genes have been discussed individually and in terms of their known ontological processes. Gene categories shown above indicated that MPP^+^-induced toxicity might be related to the production of oxidative stress, DNA and protein damage, cell growth arrest, cell cycle/proliferation repression and apoptosis. IRIDESCENT was used to gain a broader perspective on how the 59 responding genes with known functions were related and whether these gene expression changes resonated with MPTP-induced neurotoxicity and Parkinson's disease. Only 47 of these 59 gene names, however, appeared in the literature at least once and could be used in the analysis based on the IRIDESCENT search. Genes with sequence similarity to other genes were not used (e.g. the LOC* entries). When examining how each of these genes was related to one another within the scientific literature, 34 genes had no detected relationships, suggesting that at least a few of these genes may be the result of noise or a non-specific response. It is also possible, of course, that their relationship to MPP^+ ^and PD has not yet been documented and are thus valuable leads.

**Figure 5:**
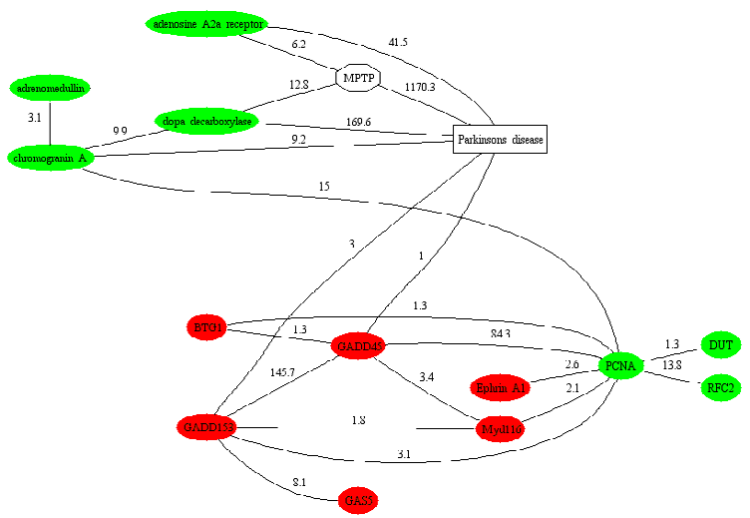
Within the subset of genes expressed at a 2-fold or greater level in the MPTP microarray experiments, these 13 were found co-mentioned together within MEDLINE abstracts at least twice. Green colors = down-regulated genes, Red = upregulated genes. Relationships with MPTP (octagon) and Parkinson's Disease (rectangle) are also displayed. Relationship weights were calculated as (number of abstract co-mentions × 0.5) + (number of sentence co-mentions × 0.8). Relationships with weights ≤ 1 are not shown.

Discarding the genes without known connections, the most apparent pathway identified by the literature analysis was the DNA damage pathway (Figure 5), with GADD45 and GADD153 (Ddit3) in a relatively central role. Interestingly, these genes have been documented in other microarray experiments to play a role in dopamine-induced toxicity [75], yet have been only weakly associated with PD thus far. Both MPTP and PD are included in this graph so that literature relationships to the responding genes are apparent. Note the strong relationship between MPTP and PD and that the genes related to MPTP are also strongly related to PD. Roughly, the graph also segregates between a responding (up-regulated) and repressed (down-regulated) group of genes. Because we expect genes related by function or process in the literature to behave in a similar manner to a greater degree than unrelated genes, this helps confirm the specificity of the microarray experiments conducted. This graph also helps visualize to a greater degree the central players in the transcriptional response – in this case, PCNA, which has been called the "ringmaster of the genome" and can lead to apoptosis when downregulated [76]. GADD45 is also upregulated, which is known to occur after DNA damage [77]. Collectively, these factors within this analysis suggest that the MOT is likely due to DNA damage. As part of the shared relationship analysis, IRIDESCENT also identified Methyl Methanesulfonate (MMS) as being highly related to the responding genes. Because MMS is known to induce DNA damage through DNA alkylation, it would be interesting to compare the MPTP and MMS responses in future microarray experiments to identify both similarities and differences in their gene expression patterns.

## Discussion

During the last decade, DNA microarrays have been one of the hottest topics in biological research and biotechnology industry. The first paper about a DNA microarray was published from Pat Brown's laboratory in Stanford University in 1995 [78]. By the end of 2004, the number of microarray-related publications was 11,625 (, keyword searching: DNA microarray). However, data assurance and data quality are still great challenges in microarray studies [4,79]. In this study, the experiment was performed under stringent quality control, with minimum standards sets for RNA purity, RNA degradation, and dye-incorporation ratios. The correlation ratio between the two sample-sets was up to 0.9, thus, we assume that variance introduced by the experimental procedure was small. The anti-correlation effect exists more or less in all dye-swapped microarray experiments, which is due to natural differences between the Cy5 and Cy3 dyes. As our data indicated, the anti-correlation was intensity-dependent, and occurred primarily with low-intensity spots. The cut-off threshold applied in this study (log intensity ≥ 8) can, therefore, largely exclude these low intensity spots. This cutoff may cause false-negative discovery because the low-expressed genes, which may be altered by treatment, are also eliminated. However, it was interesting to note that none of the genes with expression levels lower than the cutoff level were changed up to 2-fold with MPP^+ ^treatment. This is mainly due to the dye anti-correlation effect as shown in Figure 2.

In this study, the good correlation of sample set 1 and sample set 2 (Fig. 4) indicated low variance, which made it justifiable to use fold-change criterion to determine genes that were altered following MPP^+ ^treatment. Actually, the correlation ratio of the two sample sets was up to 0.98 if only those genes with 2-fold change were counted (data not shown). Two-fold is a somewhat arbitrary fold-change criterion that has been applied in many microarray studies [80-83], but nonetheless necessary to delineate a tentative set of responders. We have to admit that it is an arbitrary criterion, and it does not mean that the genes altered lower than 2-fold change are not meaningful. Certainly, more genes will be determined to be significant if the threshold is decreased to 1.5 or lower, but this will produce more false discovery. Currently, this criterion is still the most acceptable criterion regarding the magnitude of fold change and biological significance.

Data interpretation (mechanism of toxicity) is the ultimate and most complicated aspect of a microarray study, in part because there is no standard approach. Although there are more and more tools, websites and biological libraries to assist MOT analysis, biological knowledge and understanding the pertinent literature are generally more important for MOT analysis. Our study indicated that MPP^+ ^may produce its toxicity via the production of oxidative stress as indicated by induction of associated genes (Vmp1, Hspa5, Herpud1, Ftl1, Efna1, Ctsl, etc.), and at the same time suppression of general defense systems (Gstm1, Cd24, Adora2a, etc.). Previous studies have demonstrated that MPP^+ ^elicits its toxicity in PC12 cells via the production of oxidative stress [84,85], and this study provides evidence of oxidative stress production at the gene level. Oxidative stress may be responsible for the DNA and protein damage as represented by the induction of DNA and protein damage-inducible genes. It has been reported that MPP^+ ^elicits DNA damage and subsequent apoptosis [86,87] and induced heat shock proteins such as HSP70, which is an indicator of protein damage and repair [88,89]. DNA and protein damage are involved in cell growth arrest and cell cycle repression and eventually apoptosis [90,91]. A plethora of evidence has demonstrated that MPP^+ ^can elicit cell death in PC12 cells [34,36,88]. Therefore, this study confirmed the previous findings at the gene expression level, and more importantly the genes identified in this study suggested specific mechanistic pathways of MPP^+^-induced toxicity.

In summary, this study provides new insight on the mechanism of toxicity induced by MPP^+^. Our data indicate that MPP^+ ^induces oxidative stress, elicits damage in DNA and proteins, and causes cell growth arrest, cell cycle suppression and apoptosis. Literature analysis by IRIDESCENT has revealed some genes such as PCNA and GADD45 that are likely central players in this toxic response. In addition, IRIDESCENT analysis demonstrated that some of the genes altered by MPP^+ ^in PC12 cells are involved in MPTP induced neurotoxicity in mice and Parkinson's disease. We have also identified MMS as a compound related to the genes in our experimental transcriptional response, and since no studies on the effects of MMS on PC12 cells have been published thus far, this is a potentially promising avenue for future experimentation the mechanism of toxicity for MPTP.

## Figures and Tables

**Table 1:** Genes associated with oxidative stress

**UniGene ID**	**Genbank Acc ID**	**Gene Symbol**	**Gene Name**	**Fold change**
Rn.840	NM_138839	Vmp1	vacuole Membrane Protein 1	3.18
Rn.10656	NM_138863	Ltb4dh	leukotriene B4 12-hydroxydehydrogenase	-4.99
Rn.22509	BF398773	isg12(b)	putative ISG12(b) protein	3.76
Rn.11088	NM_013083	Hspa5	heat shock 70 kD protein 5	4.03
Rn.4028	NM_053523	Herpud1	homocysteine-inducible, endoplasmic reticulum stress-inducible, ubiquitin-like domain member 1	2.56
Rn.93760	NM_017014	Gstm1	glutathione S-transferase, mu 1	-2.60
Rn.1905	NM_022500	Ftl1	ferritin light chain 1	2.85
Rn.8427	BF284120	Efna1	ephrin A1	2.03
Rn.6007	NM_012752	Cd24	CD24 antigen	-3.80

**Table 2:** Genes associated with DNA and protein damage

**UniGene ID**	**Genbank Acc**	**Gene Symbol**	**Gene Name**	**Fold change**
Rn.2232	NM_133546	Myd116	myeloid differentiation primary response gene 116	3.50
Rn.6102	NM_053592	Dut	Deoxyuridinetriphosphatase (dUTPase)	-2.46
Rn.11183	NM_024134	Ddit3	DNA-damage inducible transcript 3	3.57
Rn.14939	BI286828	Wars	tryptophanyl-tRNA synthetase	2.12
Rn.7102	AI412322	Tcp1	t-complex protein 1	-2.06
Rn.3102	AI102920	LOC296368	similar to ubiquitin-conjugating enzyme E2C; DNA segment, Chr 2, ERATO Doi 695, expressed	-2.04
Rn.11088	NM_013083	Hspa5	heat shock 70 kD protein 5	4.03
Rn.1294	NM_013156	Ctsl	cathepsin L	2.89

**Table 3:** Genes associated with metabolic process

**UniGene ID**	**Genbank Acc**	**Gene Symbol**	**Gene Name**	**Fold change**
Rn.10826	NM_030834	Slc16a3	monocarboxylate transporter	-2.18
Rn.83595	NM_031841	Scd2	stearoyl-Coenzyme A desaturase 2	-2.20
Rn.874	NM_012615	Odc1	ornithine decarboxylase 1	-2.22
Rn.29938	AI409214	LOC360688	similar to glucan (1,4-alpha-), branching enzyme 1; DNA segment, Chr 16, ERATO Doi 536, expressed	-5.38
Rn.37838	NM_022922	LOC300465	similar to triosephosphate isomerase 1	-2.83
Rn.3687	NM_017025	Ldha	lactate dehydrogenase A	-3.54
Rn.64496	NM_023964	Gapds	glyceraldehyde-3-phosphate dehydrogenase, spermatogenic	-2.18
Rn.28161	NM_053445	Fads1	fatty acid desaturase 1	-2.28
Rn.41024	NM_021655	Chga	chromogranin A	-2.07
Rn.87821	NM_053623	Acsl4	acyl-CoA synthetase long-chain family member 4	2.15
Rn.87821	NM_053623	Acsl4	acyl-CoA synthetase long-chain family member 4	2.84

**Table 4:** Genes associated with neurotransmission and neuronal growth

**UniGene ID**	**Genbank Acc ID**	**Gene Symbol**	**Gene Name**	**Fold change**
Rn.9704	NM_030997	Vgf	VGF nerve growth factor inducible	-2.19
Rn.12100	NM_031821	Plk2	polo-like kinase 2 (Drosophila)	4.00
Rn.5785	NM_053601	Nnat	neuronatin	-2.23
Rn.14673	BI302561	LOC303601	similar to Cytochrome b561 (Cytochrome b-561)	-2.39
Rn.14673	BI302561	LOC303601	similar to Cytochrome b561 (Cytochrome b-561)	-2.04
Rn.2874	NM_017187	Hmgb2	high mobility group box 2	-2.23
Rn.11064	NM_012545	Ddc	dopa decarboxylase	-2.79
Rn.11064	NM_012545	Ddc	dopa decarboxylase	-2.68
Rn.10784	NM_133303	Bhlhb3	basic helix-loop-helix domain containing, class B3	-2.34

**Table 5:** Genes associated with cell growth arrest and apoptosis

**UniGene ID**	**Genbank Acc ID**	**Gene Symbol**	**Gene Name**	**Fold change**
Rn.22325	NM_144755	Trib3	tribbles homolog 3 (Drosophila)	4.66
Rn.14733	BI303289	Gas5	growth arrest specific 5	2.47
Rn.10250	NM_024127	Gadd45a	growth arrest and DNA-damage-inducible 45 alpha	2.19
Rn.8427	BF284120	Efna1	ephrin A1	2.03
Rn.840	NM_138839	Vmp1	vacuole Membrane Protein 1	3.18
Rn.11180	NM_053294	Adora2a	adenosine A2a receptor	-2.31
Rn.10232	NM_012715	Adm	adrenomedullin	-2.13

**Table 6:** Genes associated with cell-cycle

**UniGene ID**	**Genbank Acc ID**	**Gene Symbol**	**Gene Name**	**Fold change**
Rn.14867	AI103327	Tcf19	transcription factor 19	-2.19
Rn.42905	NM_021693	Snf1lk	SNF1-like kinase	-2.05
Rn.3233	NM_053485	S100a6	S100 calcium binding protein A6 (calcyclin)	-2.09
Rn.22905	AA899195	Rfc2	replication factor C (activator 1) 2 (40 kD)	-2.14
Rn.22905	AA899195	Rfc2	replication factor C (activator 1) 2 (40 kD)	-2.14
Rn.44178	AJ299016	Ret	ret proto-oncogene	-3.61
Rn.223	NM_022381	Pcna	proliferating cell nuclear antigen	-2.41
Rn.12072	NM_012603	Myc	v-myc avian myelocytomatosis viral oncogene homolog	2.02
Rn.33226	U17565	Mcmd6	mini chromosome maintenance deficient 6 (S. cerevisiae)	-2.54
Rn.41428	AI706769	LOC304951	similar to NUF2R protein	-2.10
Rn.8884	AI112987	LOC292071	similar to CDT1 protein	-2.26
Rn.3477	AA858962	LOC287280	similar to S-phase kinase-associated protein 1A isoform b; organ of Corti protein 2; transcription elongation factor B (SIII), polypeptide 1-like; RNA polymerase II elongation factor-like protein OCP2; cyclin A/CDK2-associated p19	-2.74
Rn.57	NM_019904	Lgals1	lectin, galactose binding, soluble 1	-2.18
Rn.3636	NM_022674	H2afz	H2A histone family, member Z	-2.23
Rn.7947	BI298478	Copeb	core promoter element binding protein	2.01
Rn.11252	NM_031642	Copeb	core promoter element binding protein	2.08
Rn.11252	NM_031642	Copeb	core promoter element binding protein	2.72
Rn.6934	NM_019296	Cdc2a	cell division cycle 2 homolog A (S. pombe)	-2.50
Rn.1000	NM_017258	Btg1	B-cell translocation gene 1, anti-proliferative	2.17
Rn.1000	NM_017258	Btg1	B-cell translocation gene 1, anti-proliferative	2.20

**Table 7:** Genes with unknown functions

**UniGene ID**	**Genbank Acc**	**Gene Symbol**	**Gene Name**	**Fold change**
Rn.6738	AA924324	LOC305709	similar to transcription elongation factor IIS – mouse	2.33
Rn.3494	NM_019144	Acp5	acid phosphatase 5, tartrate resistant	-3.36
Rn.13778	NM_133298	Gpnmb	glycoprotein (transmembrane) nmb	2.69
Rn.24007	AI178104	LOC293623	similar to RIKEN cDNA 2400009B11 gene	-2.10
Rn.1935	BI288713	LOC305234	similar to genethonin 1	2.94
Rn.7233	AI230347	Ns5atp9	Ns5atp9 protein	-4.04
Rn.18835	AW252093	LOC360847	similar to RIKEN cDNA 2700084L22	-2.05
Rn.12866	BQ782988	LOC361168	similar to HTPAP protein	2.32
Rn.18387	AW251335	LOC363028	similar to RIKEN cDNA 2410030K01	-2.21
Rn.22267	AA943981	LOC311209	similar to endoplasmic reticulum membrane protein with at least 3 transmembrane domains of bilaterial origin like (XB300)	-2.05
Rn.8733	NM_138892	LOC300708	similar to RS21-C6 protein	-2.37
